# A New Breathing Technique for Pain Management in Patients With Thoracic Trauma: A Randomised Trial

**DOI:** 10.1002/pri.70152

**Published:** 2025-12-19

**Authors:** Sameer Tootla, Monika Fagevik Olsén, Jenny Danielsbacka, Heleen van Aswegen

**Affiliations:** ^1^ Department of Physiotherapy School of Therapeutic Sciences Faculty of Health Sciences University of the Witwatersrand Johannesburg South Africa; ^2^ Department of Neuroscience and Physiology/Health and Rehabilitation Gothenburg University Gothenburg Sweden; ^3^ Department of Physiotherapy Sahlgrenska University Hospital Gothenburg Sweden

## Abstract

**Background:**

Pain management following thoracic trauma is important for optimal patient outcomes. One nostril inspiration (ONI) encourages slow inspiration through one nostril to minimise inspirational flow, avoiding rapid breathing movements which induce pain.

**Objectives:**

To investigate the effects of ONI, added to standard physiotherapy management, on patients' reported pain levels, and on functional status, exercise capacity, pulmonary complications, adverse events, and hospital length of stay (LOS).

**Methods:**

A single‐centre randomised trial was conducted. Adults with confirmed thoracic trauma, spontaneous breathing, and able to cooperate were recruited using consecutive sampling. Participants in two trauma wards received either standard physiotherapy or experimental care (standard physiotherapy plus ONI). A computer‐generated randomisation list was used to determine ward allocation for standard (SC) and experimental care (EC). Allocation was 1:1. EC participants performed sets of 10 ONI breaths every second waking hour and when experiencing pain. Training was recorded in a diary. Outcomes were assessed on days 1–5 of admission. Descriptive and inferential analyses were performed.

**Results:**

Participants (*n* = 150; median age 31 (IQR 26–39) years; 92.7% male) had mostly sustained penetrating thoracic trauma from physical assault (82.7%). Most participants had intercostal drains in place. Both groups received a median of four physiotherapy sessions. EC participants performed ONI breathing at a median frequency of 2.8 (IQR: 2.1–3.6) times/day; 45% adhered to ≥ 75% of prescribed sessions. Pain decreased in both groups. On day 5, EC reported significantly lower pain (*p* = 0.01, moderate effect size *d* = 0.72). Participants practicing ONI ≥ 3 times/day had less pain on day 3 than those with lower adherence (*p* = 0.05). Secondary outcomes showed a slightly longer mean LOS for EC (5.0 vs. 4.5 days, *p* = 0.008). No between‐group differences were found for pulmonary complications, functional or exercise capacity, or adverse events. All participants were discharged home.

**Conclusion:**

Education on repeated slow deep breathing exercise taught to patients with thoracic injury reduced pain, with ONI being one method of slow deep breathing. Those who adhered closely to the protocol had reduced pain from the third day of hospitalisation. ONI is a feasible and safe adjunct to standard physiotherapy that empowers patients to manage their respiratory pain.

**Trial Registration:**

The study was registered with the Pan African Clinical Trials Registry (PACTR202201706227234)

**Abbreviations**
EC: Experimental care
FSS‐ICU: Functional status score in intensive care
ICD: Intercostal drainage
LOS: Length of stay
MVA: Motor vehicle accident
ONI: One nostril inspiration
PVA: Pedestrian vehicle accident
SA: South Africa
SC: Standard care
TUG: Timed up and go

## 1 Background

Traumatic injury remains a main contributor to morbidity and mortality globally (World Health Organisation 2024) and for citizens of South Africa (SA) (Prinsloo et al. 2024). Up to 60% of traumatic injuries include thoracic trauma (Dogrul et al. 2020). These injuries are sustained through blunt or penetrating mechanisms such as road traffic accidents due to reckless driving and use of non‐roadworthy vehicles, and interpersonal violence because of continued socio‐economic inequality, or alcohol and substance abuse (Prinsloo et al. 2024). All cases of thoracic injury are admitted to the emergency care unit for evaluation through advanced trauma life support strategies. Those with moderate to severe injury are admitted to the intensive care unit or trauma ward for monitoring and management (Van Aswegen, Roos, Svensson‐Raskh, et al. 2025).

Conservative management of patients with thoracic trauma involves placement of an intercostal drainage (ICD) system to manage pneumothorax, aemothorax or a combination of these, to evacuate retained air and blood from the interpleural space (Van Aswegen, Roos, Svensson‐Raskh, et al. 2025). Standard physiotherapy rehabilitation for such patients includes deep breathing exercises, active coughing, active exercises to encourage trunk mobility and upper limb movements, and early mobilisation to maintain functional activity within the ward setting (Fagevik Olsén et al. 2025).

Acute respiratory pain is a common complaint of patients with thoracic injury and influences their physical function abilities (Weinberg et al. 2022). Acute pain results from soft tissue damage on the chest wall, pleural pain due to irritation or inflammation of the parietal pleura during insertion of the ICD tubing, size and positioning of the ICD tubing within the thorax, and chest wall instability from rib or sternal fractures (Lee et al. 2012; Mergner 2017; Van Aswegen, Roos, Svensson‐Raskh, et al. 2025). Intercostal nerve pain results from injury or irritation of the nerve or compression of the nerve by the ICD tubing that rests directly on the nerve (Mergner 2017), and is exacerbated by movement, coughing, or palpation of the chest wall and can persist beyond the initial post‐procedural period (Lee et al. 2012). Patients with acute pain are often unwilling to breathe deeply, cough effectively, move their trunk and upper limbs, and mobilise out of bed (Van Aswegen, Roos, Haarhoff, et al. 2025). This increases their risk of developing complications such as lung atelectasis, pneumonia, trunk stiffness, and decreased functional capacity.

Well‐controlled pain management is important to promote physical activity and optimal patient outcomes following thoracic trauma (Pharaon et al. 2015; Flarity et al. 2017; Kim and Moore 2020). Pain management includes pharmacological therapy combined with non‐pharmacological approaches (Weinberg et al. 2022). One type of non‐pharmacological pain management approach used by physiotherapists is education of patients on how to support their chest wall around the insertion site of the ICD system tube, and the area of the fractures, to reduce pain when performing deep breathing, and to facilitate a stronger cough effort (Van Aswegen et al. 2020).

Another non‐pharmacological pain management strategy is slow deep breathing. Slow deep breathing may decrease acute pain intensity (Joseph et al. 2022). This study presents and evaluates a new breathing intervention, one nostril inspiration (ONI). ONI is a simple breathing method to teach and easy for patients to learn. It assumes that slow inspiration through one nostril (the other being closed by hand) minimises the inspirational flow and rate of breathing, hence avoiding rapid breathing movements which induce pleuritic pain. This breathing technique makes it possible for patients to take larger than tidal volume breaths in a slow and more controlled way, and for the patient through these controlled breaths, being able to avoid shallow breathing which could lead to pulmonary complications. ONI was developed in clinical settings for patients with different kinds of thoracic pain (e.g., pulmonary embolism) but not tested previously in those with thoracic injury. The clinical results observed in other populations highlight that patients who receive a way to control and master their pleuritic pain, rather than being disabled by it, have a greater satisfaction regarding pain control and less fear of breathing deeply or active coughing.

No evidence exists to describe the effect of ONI, as a non‐pharmacological pain management strategy, on the clinical outcomes of patients with thoracic injury. ONI does not require any additional equipment and is being evaluated in this study for its ease of use and low cost to apply to patient care in trauma centres in middle‐ and low‐income areas. It is thus appropriate to test its potential effects on patient outcomes in a public healthcare sector hospital situated within a middle‐income country. The rationale for this study was primarily to investigate the effects of ONI, added to standard physiotherapy management of patients with thoracic trauma, on their reported level of pain, and secondarily on functional status, exercise capacity, pulmonary complications, adverse events, and hospital length of stay (LOS).

## 2 Methods

### 2.1 Study Design and Setting

This single‐centre, single blinded randomised trial was performed at a university‐affiliated public sector hospital from April 2022 to the end of May 2023. Reporting was done using the CONSORT statement and checklist. This study forms part of a larger project titled ‘Physiotherapy management of patients who sustained thoracic trauma: a multi‐centre study’. The larger study is a collaboration between trauma centres in SA and Sweden. This study is Phase 2 of the project and includes data only from the SA cohort.

### 2.2 Study Participants

Patients who suffered thoracic trauma and were admitted to the two trauma wards, were screened for possible participation and recruited using a consecutive sampling technique. Inclusion criteria were adults (≥ 18 years), diagnosed with thoracic injury, breathing spontaneously and who could cooperate (Standardised 5 questions score of 4–5/5) regarding the interventions. Exclusion criteria were accompanying acute or previously diagnosed spinal cord injury or traumatic brain injury, fractures of the pelvis or lower limbs that restricted active mobilisation, amputation of the lower limb/s, accompanying extensive abdominal trauma (repeated laparotomy procedures) that required prolonged immobilisation, or extensive data missing from the data capturing forms.

The chief physiotherapist was responsible for recruiting participants for the study and screened the wards daily for new admissions. On receipt of participants' written consent after being given written and oral information, the chief physiotherapist assigned each participant to either the standard care (SC) group or the experimental care (EC) group according to the ward they were recruited from. One of the researchers and the chief physiotherapist determined prior to participant recruitment, which ward would be allocated to EC and which ward would continue with SC. Ward allocation was alternated in terms of EC or SC to accommodate the different types of patients in both groups. A computer‐generated randomisation list was used to determine ward allocation with an allocation sequence 1:1. The list was kept in a file locked in the chief physiotherapist's office. Randomisation of wards was repeated every 3 weeks until the end of participant recruitment.

**FIGURE 1:**
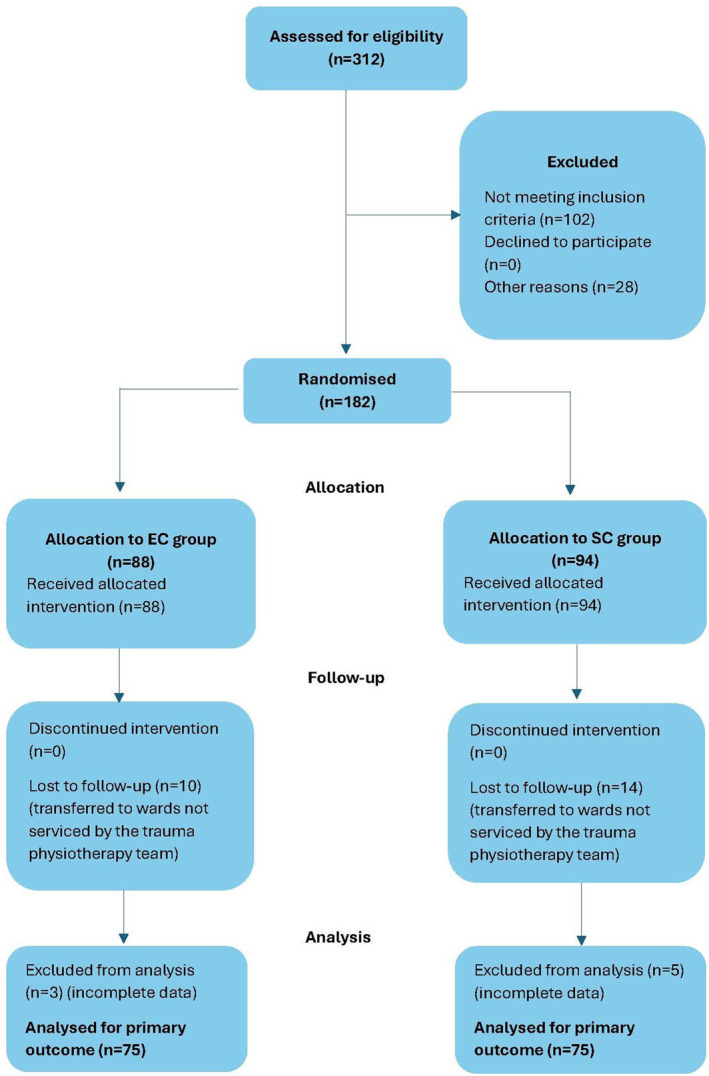
Flow chart summary of participant recruitment for the study.

The number of participants admitted into the two trauma wards during the study period and the participant recruitment to the study is summarised in Figure 1.

### 2.3 Interventions

At the participating hospitals, the standard pharmacological management of pain experienced due to thoracic injury consists of oral administration of tramal and paracetamol.

All participants received standard physiotherapy management of patients with thoracic trauma at the participating hospital. Based on individual patient needs, management typically consists of breathing exercises (e.g., deep breathing exercises, active cycle of breathing technique, positive expiratory pressure (glove blowing)), patient education about repetition of breathing exercises on their own (at minimum three times daily), mobilisation away from the bedside, cycling on a stationary bicycle, shoulder and trunk range of motion exercises and education on pain management. The education provided includes the importance of breathing exercises and its effects on pain management and improvement in pulmonary function. It also includes the importance of early mobilisation and the benefits of exercise therapy. The interventions were performed once daily and supervised by the treating physiotherapist. An exercise programme was given to each patient to follow when the physiotherapist left for the day.

The participants in the EC group, in addition to receiving standard physiotherapy management, were also taught to use ONI to assist in managing their pain when breathing. ONI consists of slow and deep inspirations through only one nostril. The other was closed by hand. The participant was informed to perform sets of 10 ONI breaths every second waking hour of the day and, in addition, when experiencing pain. Their training was recorded in a diary. They were expected to perform ONI eight times per day according to the diary provided. Re‐enforcement of the ONI technique was done each day by the treating physiotherapist reminding the participant to continue the technique and to document every 2 hours when it was done in the ONI diary.

### 2.4 Outcomes

The primary outcome measure for this study was pain assessed by the Wong‐Baker FACES pain rating scale (Oliveira et al. 2014). It contains drawings of six different faces illustrating pain levels from ‘no hurt’ (0) to ‘hurts worst’ (10). The scale is valid for use with adults (Kawamura et al. 2009; Van Giang et al. 2015; South African Society of Anaesthesiologists 2022).

Secondary outcomes were:

- hospital LOS (days).
- incidence of pulmonary complications during hospital stay (e.g., diagnosed pneumonia, atelectasis, respiratory distress necessitating high flow oxygen therapy or non‐invasive ventilation, respiratory failure necessitating intubation and mechanical ventilation).
- physical function assessed by Functional Status Score in Intensive Care (FSS‐ICU) for patients with acute illness. The function is assessed on a scale from ‘unable to perform (0)’ to ‘complete independence (7)’ (Huang et al. 2016).
- exercise capacity was evaluated by three tests:
  ◦ 10‐m walk test where patients walk the distance in comfortable and maximal speed. Time (in seconds) was recorded. This test is reported to be acceptable of functional ability and walking speed in older adults (Peters et al. 2013).
  ◦ Timed Up and Go (TUG) test is a test of functional balance and walking speed. From sitting, the patient raises, walks 3 m, turns, walks back and sits down. Time (in seconds) was registered. The TUG test is a widely used reliable and valid tool for the assessment of functional balance in a variety of patient populations (Steffen et al. 2002)
  ◦ 1 minute sit‐to‐stand test is a test of muscular strength and endurance (Bohannon and Crouch 2019). The patient performs as many cycles of standing ups and sitting downs as possible from a standardised chair for 1 minute.
- adverse events occurring during physiotherapy sessions (e.g., falls, dislocation of lines, dizziness or fainting, desaturation).

### 2.5 Procedures

Before recruitment started, one of the researchers met with all clinical physiotherapists at the two trauma wards to discuss the aim and objectives of the study and educated them on how ONI should be taught and administered to participants. This education was repeated when new rotational physiotherapy staff joined the physiotherapy trauma team.

The chief physiotherapist allocated each participant who consented to the trial to the clinical physiotherapists who administered either SC or ONI plus SC to each participant as per ward allocation. One of the researchers was responsible for the continued assessment of patient outcomes as per study procedure. The researcher remained blinded to participant allocation as he assessed and re‐assessed participant outcomes during the trial. The participants were informed prior to being assessed that they should not inform the assessor which group they were in to avoid any researcher bias. The researcher performed all test procedures on the participants in the afternoon on days 1, 3 and 5. Data were captured on a study‐specific data capture form.

## 3 Statistics

In Phase 1 of the project, pain was observed in patients with thoracic trauma in the SA cohort (Van Aswegen, Roos, Haarhoff, et al. 2025). The average pain on day one was 4 on the Wong Baker FACES pain scale. To decrease pain at one level between the groups (SD 2), a sample size of 63 patients per group was necessary (alpha set at 0.05, power at 0.80 (beta at 0.20). Taking into consideration non‐compliance and dropouts, 75 patients were recruited per group.

Data captured in Excel was imported into IBM SPSS Statistics version 29 software to assist with statistical analysis. Intention‐to‐treat analysis was performed. Descriptive statistics were used to present demographics and results as number, percentage, means and standard deviations (SD) for normally distributed data, and medians and interquartile ranges (IQR) for non‐normally distributed data. Chi‐squared test and independent *t*‐test were used to perform between group comparisons. Chi‐squared test was also used for within group analysis in the experimental group. Throughout testing was done at the 5% level of significance (*p* ≤ 0.05). Cohen's *d* was calculated to determine the magnitude of the between‐group differences in reported levels of pain on days 1, 3 and 5 of hospitalisation. Interpretation of effect sizes was done using *d* = 0.2 as small, *d* = 0.5 moderate, and *d* = 0.8 large effect (Lakens 2013; Sullivan and Feinn 2012). Pearson correlation was performed to test for association between the frequency of ONI performed per day and the level of pain reported on days 3 and 5 of hospitalisation in the experimental group. Interpretation of r‐values was performed according to Schober et al. (2018) guidelines.

## 4 Ethics

The study received ethics approval from the University of the Witwatersrand Human Research Ethics (Medical) committee (certificate number: M200222). Permission to conduct this project was obtained from the National Health Research Database Gauteng province on 21 August 2020. This protocol was registered on the Pan African Clinical Trials Registry (PACTR202201706227234). The ethical principles related to conduct of research on human subjects as outlined in the Declaration of Helsinki were observed for the duration of this trial.

## 5 Results

The median age for the total cohort was 31 (IQR: 26–39) years with minimum age of 18 and maximum age of 50 years (Table 1). The majority of participants were male (*n* = 139, 92.7%) and less than half of the total cohort were smokers (*n* = 60, 40%). Most participants in both groups sustained penetrating trunk trauma (Table 1).

**TABLE 1:** Baseline characteristics of the participants. Median (IQR) or *n* (%).

Variable	Total	Experimental group (*n* = 75)	Standard treatment (*n* = 75)	*p*‐value
Age, years	31 (26–39)	34 (26–41)	30 (26–37)	0.054
Height, m	1.71 (1.64–1.74)	1.7 (1.63–1.72)	1.71 (1.65–1.74)	0.156
Weight, kg	68 (61–72)	67 (60–71)	68 (61–74)	0.253
Body mass index, kg/m^2^	23 (21.9–24.9)	23 (21.8–25.1)	23 (22.5–24.9)	0.793
Sex (*n*, %)				
Male	139 (92.7)	71 (94.7)	68 (90.7)	0.347
Female	11 (7.3)	4 (5.3)	7 (9.3)	
Smoking status (*n*, %)				
Non‐smoker	90 (60)	42 (56)	48 (64)	0.317
Smoker	60 (40)	33 (44)	27 (36)	
Type of injury (*n*, %)				
Blunt	30 (20)	17 (22.7)	13 (17.3)	0.414
Penetrating	120 (80)	58 (77.3)	62 (82.7)	
Mechanism of injury (*n*, %)				
Assault	124 (82.7)	58 (77.3)	66 (88)	0.245
Attempted suicide	2 (1.3)	1 (1.3)	1 (1.3)	
MVA	22 (14.7)	14 (18.7)	8 (10.8)	
PVA	2 (1.3)	2 (2.7)	0 (0)	
Chest wall injury (*n*, %)				
No rib fractures	120 (80)	57 (76)	63 (84)	0.496
1–3 ribs fractured left side	23 (15.3)	14 (18.7)	9 (12)	
1–3 ribs fractured right side	6 (4)	3 (4)	3 (4)	
> 3 ribs fractured bilaterally	1 (0.7)	1 (1.3)	0	
Intrapleural abnormality (*n*, %)				
Unilateral pneumothorax	82 (54.7)	45 (60)	37 (49.3)	0.188
Unilateral haemo‐/haemopneumothorax	64 (42.7)	27 (36)	37 (49.3)	
Bilateral haemo‐/haemopneumothorax	4 (2.7)	3 (4)	1 (1.3)	

Abbreviations: MVA, motor vehicle accident; PVA, pedestrian vehicle accident.

None of the participants reported having a chronic respiratory disease prior to hospital admission (data not shown). The mechanism of injury was most often assault (82.7%). In both groups, most participants had no rib fractures and unilateral pneumothorax were the most common intrapleural abnormality. The two groups were comparable at baseline.

One participant had a pulmonary contusion, and another had a diaphragm rupture, both in the EC group.

Regarding other injuries sustained in addition to thoracic trauma, one in each group had an upper limb fracture and one in each group an internal organ injury (both *p* = 0.316). There were no significant between‐group differences for other injuries sustained.

Forty‐three percent of the participants (*n* = 64) received some form of analgesia at the time of the first physiotherapy assessment. None of the participants received any type of sedation or received any oxygen supply. All but one participants had an ICD system in place (*n* = 149, 99.3%). Most had a unilateral ICD (*n* = 144, 96%) and five participants had bilateral ICD in place (EC group: *n* = 3, 4%; SC group: *n* = 2, 2.7%). Two patients in the EC group needed additional surgery and underwent a laparotomy (*n* = 1 for splenic injury; *n* = 1 for diaphragm rupture). None of the participants underwent rib fixation surgery.

### 5.1 Physiotherapy Treatment Received and Adherence to ONI Training

The number of physiotherapy contact sessions during hospital stay for participants in the EC group was, in median, 4 (IQR: 3–5) and SC group was 4 (IQR: 3–4). EC group participants performed ONI at a median frequency of 2.8 (IQR: 2.1–3.6) times per day whilst they were in the hospital. The minimum and maximum frequency of ONI breathing per day was 1.7 and 5.7 times per day respectively. Forty‐five percent (*n* = 34) of these patients completed 75% or more of the training sessions between the initial session and their discharge.

### 5.2 Primary Outcome—Pain

Participants reported their level of pain experienced at rest on days 1, 3 and 5 of hospitalisation (Table 2).

**TABLE 2:** Comparisons of pain levels experienced at rest measured on days 1, 3 and 5 of hospitalisation.

	Mean (SD)	Mean difference	*p*‐value	Cohen's *d*	95% CI
Day 1					
Experimental group (*n* = 74)	5.5 (1.8)	0.08	0.38	0.05	−0.27; 0.37
Standard treatment (*n* = 75)	5.6 (1.6)				
Day 3					
Experimental group (*n* = 75)	3.5 (1.9)	0.18	0.28	0.09	−0.23; 0.41
Standard treatment (*n* = 73)	3.7 (1.8)				
Day 5					
Experimental group (*n* = 30)	2.1 (1.6)	1.09	0.01	0.72	0.12; 1.3
Standard treatment (*n* = 19)	3.1 (1.3)				

Participants in both groups reported a gradual decline in pain intensity over the first 5 days of hospitalisation. Between‐group analysis showed that on days one and three, there were no significant differences in reported levels of pain at rest between the two groups. Several participants in both groups had been discharged home before day 5 of hospitalisation. Baseline characteristics (age, sex, type of injury, type of chest wall injury, intrapleural abnormality, and site of ICD insertion) for the remaining participants were re‐analysed to test for heterogeneity. No heterogeneity was detected for age (*p* = 0.06), sex (*p* = 0.30), type of injury (*p* = 0.36), type of chest wall injury (*p* = 0.08), intrapleural abnormality (*p* = 0.69), and site of ICD insertion (*p* = 0.42). On day 5 of hospitalisation, the remaining participants in the SC group reported significantly higher pain levels at rest than those remaining in the EC group (*p* = 0.01). The point estimate of 0.72 suggests a moderate effect of ONI breathing on the EC participants' level of pain.

Within‐group analysis showed that those who performed ONI breathing less than three times per day reported higher pain levels on day 3 compared to those who performed ONI three or more times per day (*p* = 0.05). There was a weak association between frequency of ONI breathing per day and level of pain reported on day 3 (*r* = 0.383) favouring the group who performed ONI more than three times per day (Wong Baker Faces pain score < 4; *p* = 0.02). No significant association between these variables was found on day 5.

### 5.3 Secondary Outcomes

The mean LOS for the EC participants was 5 (SD 1.3) days and 4.5 (SD 1.1) days in the SC group 1 (*p* = 0.008).

None of the participants in the two groups developed the predetermined pulmonary complications.

There were no significant differences in the functional status or exercise capacity (Table 3) between the groups. There were, however, gradual improvements throughout the days of assessment in both groups.

**TABLE 3:** A comparison of functional outcomes and exercise capacity assessed between groups on days one to five of hospital stay.

Variable	Day 1			Day 3			Day 5		
	Standard treatment (*n* = 75)	Experimental group (*n* = 75)	*p*‐value	Standard treatment (*n* = 63)	Experimental group (*n* = 66)	*p*‐value	Standard treatment (*n* = 19)	Experimental group (*n* = 30)	*p*‐value
10m‐walk test (m/s) median (IQR)									
Comfortable pace	1.21	1.21	0.592	1.3	1.28	0.427	1.28	1.3	0.841
	(1.2–1.28)	(1.2–1.28)		(1.23–1.31)	(1.22–1.31)		(1.24–1.3)	(1.24–1.34)	
Fast pace	1.51	1.51	0.630	1.57	1.54	0.317	1.59	1.57	0.430
	(1.45–1.59)	(1.47–1.58)		(1.48–1.62)	(1.48–1.61)		(1.46–1.62)	(1.5–1.64)	
TUG (seconds) median (IQR)	12.3	12.3	0.263	11.45	11.4	0.291	11.4	11.35	0.568
	(12.1–13.1)	(12.1–13.12)		(11.1–12.3)	(11.1–12.3)		(11.1–12.1)	(10.9–12.15)	
Sit‐to‐stand in 1 minute (repetitions) median (IQR)	17	16	0.6	24	21	0.597	19	21	0.109
	(12–22)	(11–19)		(12–26)	(12–25)		(14–24)	(16–24)	

Abbreviation: TUG, timed up and go.

None of the participants presented with any prior walking impairments at recruitment to the study or needed physical assistance to complete any of the included tests.

There were no reports of any adverse events that occurred during physiotherapy sessions in either group. No participant deaths were reported during the trial.

All the patients were discharged home. The treating physiotherapists did not deem it necessary for any of the patients to receive out‐patient physiotherapy follow‐up after discharge.

## 6 Discussion

Penetrating trauma due to assault was the most common cause of thoracic injury in young adults who participated in this study, with a male predominance. Male predominance is comparable to international (Lundin et al. 2022; World Health Organisation 2024) and SA reports (Kithuka et al. 2023; Lüttich et al. 2025; Van Aswegen, Roos, Haarhoff, et al. 2025) on gender‐related incidence of trauma. Among young adults in SA, particularly those younger than 35 years, thoracic injuries are mostly associated with high‐energy mechanisms, such as motor vehicle accidents and violent encounters (Prinsloo et al. 2024). This age group is generally more active and engages in riskier behaviours, leading to a higher likelihood of sustaining traumatic injury (Prinsloo et al. 2024). Additionally, lifestyle factors such as smoking, alcohol consumption and substance abuse contribute to the prevalence and severity of traumatic injury (Sommer et al. 2017). Assault as the main mechanism of injury resonates with the increase in assaults reported to the police services in SA during 2021/2022 (Statistics South Africa 2022). Forty‐three percent of participants received analgesia, few had rib fractures, and they were not otherwise severely injured. None were on oxygen therapy at the first physiotherapy contact. This confirms their low injury severity.

The primary outcome, pain, was found to gradually decrease in both groups during the study period of 5 days. The differences between the groups were small, but there are some tendencies between the groups. It seems like ONI may have an impact on respiratory pain, as found on day 5, as those in the SC group reported higher pain levels at rest. This finding is consistent with the systematic review by Joseph et al. (2022) that concluded that slow deep breathing has a significant effect on decreasing reported levels of acute pain in those who perform the technique. In addition, it was found that participants who used ONI more frequently during the day had a greater benefit from it as they reported little to no pain by day 3 of hospitalisation compared to those who performed ONI less than three times per day. These results must be confirmed in future trials of patients with thoracic trauma.

The ONI diary was completed by the EC participants themselves. It was found that less than half of the participants completed 75% or more of the ONI sessions. This brings about the concern for compliance with ONI breathing. There are three possible explanations for this phenomenon. Firstly, most participants sustained penetrating thoracic injury due to assault. An important cause of penetrating injury in SA, as mentioned, is intentional injury associated with lifestyle behaviours (e.g., alcohol and substance abuse) that lead to aggression and interpersonal violence and result in stab injuries (Sommer et al. 2017; Kithuka et al. 2023; Lüttich et al. 2025; Van Aswegen, Roos, Haarhoff, et al. 2025). Some participants may have suffered withdrawal symptoms during their first days of hospitalisation that affected their adherence to ONI training. Secondly, a known side‐effect of Tramal is drowsiness. Participants who experienced drowsiness may have been less inclined to adhere to ONI training. Lastly, participants' understanding of when ONI training should have been done, may have impacted their adherence. They were instructed by the treating physiotherapist to perform ONI two‐hourly and when experiencing pain. It is possible that some only performed ONI training when experiencing pain and that others could not maintain the two‐hourly training as requested, particularly if they were sleeping during the day due to withdrawal and/or drowsiness. Previously Frost et al. (2016) reported that individual diary entries to measure adherence to training may provide motivational effects for some participants. One may assume that participants were truthful with the ONI diary entries due to the daily reminders from treating physiotherapists to complete the ONI therapy and document each session in the diary.

Even if ONI may have an impact on respiratory pain, it, or less pain, did not affect the participants' functional ability or exercise capacity. None of the participants developed any pulmonary complications or had no adverse events during the physiotherapy interventions. The EC group, however, had a longer LOS by half a day compared to the SC group, which was statistically significant. Its clinical significance is debatable. Possible explanations for the longer LOS for EC participants are related to the characteristics of this group. These participants were slightly older, more were current smokers, more had laparotomy procedures, and more had rib fractures than those in the SC group.

Changes in physical function were assessed with the FSS‐ICU, 10‐m walk test and TUG test. This study is part of an international collaboration including patient cohorts of different ages and different proportions of penetrating and blunt thoracic trauma. The scores have ceiling effects and as the current cohort of patients were younger and few had severe pain at inclusion, this may have an impact on the results.

There are both limitations and strengths to this study. On days when the physiotherapy department was short staffed and had a high patient load, participant recruitment decreased leading to a prolonged period of recruitment. This was a single‐centre trial, which limits the generalisability of the results. Self‐reporting is an important part of pain assessment (South African Society of Anaesthesiologists 2022). The Wong Baker FACES pain rating scale was used to assess the level of pain in this study to ensure that participants understood what was being asked of them regarding pain intensity. South Africa is a multilingual nation, and the FACES pain scale is considered appropriate for use in this population (South African Society of Anaesthesiologists 2022). A limitation, however, is that the dosage and times of administration of analgesia to participants were not captured. This might have affected the reported pain score of participants depending on when the assessing physiotherapist asked them about their pain level. Results should be interpreted with caution. Lastly, adherence of the SC participants to performing of deep breathing exercises was not considered in the planning of the study.

This study contributes towards the United Nations Sustainable Development Goal 3 of good health and well‐being by highlighting the benefits of ONI on reducing pain in patients with thoracic trauma who can participate in this type of training.

## 7 Conclusion

Education on repeated slow deep breathing exercise taught to patients with thoracic injury helped to reduce pain early during hospitalisation, with ONI being one method of slow deep breathing. Those who adhered more closely to the prescribed ONI protocol experienced greater reductions in pain. This breathing technique is a safe adjunct to standard physiotherapy and appropriate for use in limited resource healthcare settings. Findings contribute to the increasing body of evidence supporting the value of non‐pharmacological pain management approaches used by physiotherapists in trauma care.

## 8 Implications for Physiotherapy Practice

ONI is a simple, no‐cost, easy‐to‐understand breathing method that empowers those who adhere to the prescribed breathing protocol, to manage their respiratory pain effectively. It can be implemented immediately in patient care as part of daily physiotherapy practice as no special equipment is needed to perform this breathing technique.

## Funding

National Research Foundation of South Africa (Grant number: 141963).

## Ethics Statement

Ethics clearance was received from the University of the Witwatersrand Human Research Ethics (Medical) Committee 26 June 2020 (Clearance certificate number: M200222).

## Conflicts of Interest

The authors declare no conflicts of interest.

## Data Availability

Data will be shared on reasonable request.
